# *In vivo* cardiovascular magnetic resonance diffusion tensor imaging shows evidence of abnormal myocardial laminar orientations and mobility in hypertrophic cardiomyopathy

**DOI:** 10.1186/s12968-014-0087-8

**Published:** 2014-11-12

**Authors:** Pedro F Ferreira, Philip J Kilner, Laura-Ann McGill, Sonia Nielles-Vallespin, Andrew D Scott, Siew Y Ho, Karen P McCarthy, Margarita M Haba, Tevfik F Ismail, Peter D Gatehouse, Ranil de Silva, Alexander R Lyon, Sanjay K Prasad, David N Firmin, Dudley J Pennell

**Affiliations:** National Institute of Health Research Cardiovascular Biomedical Research Unit, Royal Brompton Hospital and Imperial College, London, UK; National Institutes of Health, Bethesda, USA

**Keywords:** Diffusion tensor imaging, Hypertrophic cardiomyopathy, Cardiovascular magnetic resonance, Myocardial architecture, Laminar structure, Sheet and shear layers, Diastolic dysfunction

## Abstract

**Background:**

Cardiac diffusion tensor imaging (cDTI) measures the magnitudes and directions of intramyocardial water diffusion. Assuming the cross-myocyte components to be constrained by the laminar microstructures of myocardium, we hypothesized that cDTI at two cardiac phases might identify any abnormalities of laminar orientation and mobility in hypertrophic cardiomyopathy (HCM).

**Methods:**

We performed cDTI *in vivo* at 3 Tesla at end-systole and late diastole in 11 healthy controls and 11 patients with HCM, as well as late gadolinium enhancement (LGE) for detection of regional fibrosis.

**Results:**

Voxel-wise analysis of diffusion tensors relative to left ventricular coordinates showed expected transmural changes of myocardial helix-angle, with no significant differences between phases or between HCM and control groups. In controls, the angle of the second eigenvector of diffusion (E2A) relative to the local wall tangent plane was larger in systole than diastole, in accord with previously reported changes of laminar orientation. HCM hearts showed higher than normal global E2A in systole (63.9° vs 56.4° controls, p = 0.026) and markedly raised E2A in diastole (46.8° vs 24.0° controls, p < 0.001). In hypertrophic regions, E2A retained a high, systole-like angulation even in diastole, independent of LGE, while regions of normal wall thickness did not (LGE present 57.8°, p = 0.0028, LGE absent 54.8°, p = 0.0022 vs normal thickness 38.1°).

**Conclusions:**

In healthy controls, the angles of cross-myocyte components of diffusion were consistent with previously reported transmural orientations of laminar microstructures and their changes with contraction. In HCM, especially in hypertrophic regions, they were consistent with hypercontraction in systole and failure of relaxation in diastole. Further investigation of this finding is required as previously postulated effects of strain might be a confounding factor.

**Electronic supplementary material:**

The online version of this article (doi:10.1186/s12968-014-0087-8) contains supplementary material, which is available to authorized users.

## Background

The compact myocardium of the left ventricle (LV) of humans and other mammals shows remarkable microstructural adaptation for global contractile function. Cardiomyocytes of the subendocardial and subepicardial layers contract almost orthogonally to each other, each angled obliquely, at helix-angles (HA) up to +60° and −60° relative to the circumferential alignments of myocytes of the mid layer [1,2]. This means that myocytes contract in one layer in the same direction as the simultaneous counter-thickening of the orthogonally orientated myocytes of another layer. However, the potential conflict between these strains is resolved and enabled to contribute to wall thickening by the small scale laminar structures called sheetlets and shear layers (Table 1) [3,4].

**Table 1:** **Glossary of terms used**

**Myocyte:**	Used here for a heart muscle cell, capable of contracting lengthwise, along its principal axis. We have not used ‘fiber’, which we do not consider appropriate in relation to myocardial structure.
**Cross-myocyte:**	Directions perpendicular to the principal axis of local myocytes.
**Sheetlets:**	Laminar arrays of myocytes, as visualized in cross-myocyte histological sections of mammalian myocardium. We use ‘sheetlets’ rather than ‘sheets’ because each is typically only a few myocytes thick and is of limited individual extent before either branching or merging with adjoining sheetlets. Sheetlets are generally orientated oblique to the local wall tangent plane, and can occur in sub-populations with opposing orientations that give herringbone-like appearances in some cross-myocyte sections.
**Shear layers:**	Collagen lined fissures or interstices between adjacent sheetlets which may be important in relation to the extents and preferential directions of aqueous diffusion through myocardium over the course of a heart cycle. The fissures are thought to allow adjacent sheetlets to slide relative to one another as they collectively swivel, increasing their obliquity (transmural angle) relative to the local wall tangent plane during contraction.
**Laminar:**	An inclusive adjective referring to myocardial ‘sheetlet and shear layer’ structure.
**E1:**	The principal eigenvector of aqueous diffusion. Previous studies have shown its direction to be aligned with local myocytes.
**E2 and E3:**	Represent the greater and the least eigenvectors, respectively, of cross-myocyte diffusion in a voxel. They are orthogonal to E1 and to each other. Although E2 and E3 may represent the sheetlet and sheet-normal directions perpendicular to myocytes, it should be remembered that DTI can only measure the mean of all diffusions in any single voxel and that a voxel of 3×3×8 mm^3^ may include two or more sub-populations of sheetlets with different orientations.
**Helix angle or E1 angle (E1A):**	Angle of obliquity of E1 in the local wall tangent plane relative to the local circumferential direction.
**Transmural angle or E2 angle (E2A):**	Angle of through-wall obliquity of E2 in the local cross-myocyte plane relative to the line of intersection of this plane with the local wall tangent plane (see Figure 1).
**Isotropic diffusion:**	Diffusion that takes place with equal a freedom in all directions, i.e. spherically, on average, from any given starting point.
**Anisotropic diffusion:**	Diffusion that is constrained by anisotropic structures so that it proceeds preferentially along certain directions or planes.

Populations of sheetlets are thought to collectively swivel around angles of approximately 45° to the local wall tangent plane, their transmural angles being smaller in diastole and larger in systole (Table 1) [5]. This allows the systolic shortening across the myocytes of one wall layer, imposed by the contraction of differently aligned myocytes of other wall layers, to be accommodated and translated to enhanced wall thickening [6]. In this way, wall thickening can be attributed to sheetlet reorientation with shear layer slippage, in addition to slight systolic increase of myocyte diameter [7]. Alternating, herringbone-like patterns of obliquely opposed sub-populations of sheetlets have been demonstrated histologically in *ex vivo* studies of several regions of the LVs of different mammalian species [3,5,8,9].

*Ex vivo* cardiac Diffusion Tensor Imaging (cDTI), based on measurements of diffusion distances of water molecules in myocardium, has been found capable of interrogating myocyte and sheetlet orientations [3,8,10–12]. From the diffusion tensor of each voxel it is possible to extract a set of three eigenvectors. The direction of the principal eigenvector (E1, associated with the largest eigenvalue) has been shown to coincide with the orientations of the myocytes (frequently described as the fibre direction), when compared to histological results [4,8]. The angle between E1 and the local radial direction is commonly referred to as the helix-angle. The secondary and tertiary eigenvectors (E2 and E3) are perpendicular to each other and E1. It has been argued that E2 and E3 align with the more and the less dominant local sheetlet populations respectively [8,10–12]. The direction and magnitude of E3 then indicates the orientation and prevalence of a secondary sheetlet sub population [8]. However this can only, by definition, apply where two intravoxel sheetlet subpopulations are themselves perpendicular to each other. This ought not to be assumed, especially not at end systole and late diastole when the angles between adjoining subpopulations may be acute or obtuse [4,7,8].

The integrity and mobility of the laminar microstructures is thought to be fundamental for LV function and could be altered in cardiovascular disease. Hypertrophic Cardiomyopathy (HCM) is an autosomal dominant genetic disease, which can be caused by mutations in the genes responsible for encoding proteins of the cardiac sarcomere [13,14]. It is characterized by increased left ventricle (LV) wall thickness in the absence of increased LV afterload and is associated with diastolic dysfunction [15,16]. Histological findings include myocyte hypertrophy and disarray, with an increase of interstitial connective tissue [17–19]. However, studies of myocardial strain in HCM have shown regional contractile heterogeneity that could not be fully explained by the distribution of regional fibrosis [20].

To date, most cDTI studies have been *ex vivo*, and evidence for the phasic reorientations of laminar structures has been provided by static, explanted hearts imaged separately in contracted and relaxed states [3,21]. An exception was work by Dou et al. where 3D diffusion data was acquired *in vivo* at six different systolic trigger times [22].

We have recently described the development of a novel *in vivo* pulse sequence for cDTI which draws on technical improvements and also the benefits of imaging at 3 T. Measures derived from this technique were recently shown to be reproducible in both controls and in HCM subjects [23,24]. In this study we used this *in vivo* sequence to analyze the cross-myocyte components of diffusion, which we hypothesized would provide information on mean intravoxel sheetlet orientations and their changes between end-systole and late-diastole in controls and show abnormalities in patients with HCM.

## Methods

### Imaging protocol

This study was approved by the National Research Ethics Service. Eleven healthy controls and 11 patients with HCM were recruited and gave written informed consent. Data in systole from 6 patients was previously reported for reproducibility [24]. Imaging was performed using a 3 T scanner (Skyra, Siemens, Erlangen, Germany) with an 18 element anterior matrix coil and 8–12 elements of a matrix spine coil. After localization of the LV short and long axes, a retro-gated balanced Steady State Free Precession (bSSFP) cine sequence with a 40 ms temporal resolution was used to find the timing and duration of the subject specific end systolic and diastolic pauses, taking care to avoid the atrial kick in diastole.

In order to ensure the same myocardial region was imaged with cDTI in systole and diastole, a breath-hold spoiled gradient echo (GRE) sequence with a spatial modulation of magnetization (SPAMM) tagging pre-pulse and a 45 ms temporal resolution was used to acquire four-chamber and two-chamber views. The linear tags were separated by 16 mm and were perpendicular to the long-axis with an acquired spatial resolution of 2.1 × 1.7 mm in-plane and slice thickness of 6 mm. The displacement of the linear tag closest to the central mid-ventricular slice was manually tracked from the systolic to the diastolic phase. The positioning of the cDTI central slice was manually adjusted between the systolic and the diastolic acquisition accordingly, with the inter-slice distance kept constant.

The *in vivo* cardiac diffusion weighted stimulated echo acquisition mode (STEAM) single shot echo planar imaging (EPI) sequence used has been previously described [23]. In short, this sequence runs over 2 heart beats and assumes that myocardium returns to the same position at the same encoding times in consecutive cycles, in this work, end systole and the late diastolic pause (diastasis). In order to minimize the length of the single shot EPI readout, parallel imaging and zonal excitation were implemented. Data were acquired in a series of breath-holds to minimize the effects of respiratory motion. Each breath-hold had a duration of 18 heartbeats. The following sequence parameters were used: reference plus 6 diffusion encoding directions at b = 350 s/mm^2^ (at a heart rate of 60 beats per minute), fat saturation, TR = 2 RR intervals = 2000 ms (at a heart rate of 60 beats per minute), TE = 23 ms, BW = 2442Hz/pixel, GRAPPA [25] parallel imaging acceleration factor of 2, EPI echo train length = 22-28 readouts, depending on field of view, EPI echo-train readout duration = 11-14 ms, in plane acquired spatial resolution = 2.7 × 2.7 mm^2^, interpolated to 1.35 × 1.35 mm^2^ (k-space zero-filling by a factor of two), field of view 360 × 123-157 mm^2^ and a minimum of 8 averages (one average per breathold). Three slices with 8 mm thickness were acquired in the mid ventricular short axis with an inter-slice gap of 4 mm. No partial Fourier was used. The sequence trigger time was set to acquire cDTI data during the systolic and diastolic rest periods. Localized first and second-order shimming and frequency adjustment were performed over the extent of the LV within the imaging planes. It should be noted that the sequence runs a spoiler gradient in place of the diffusion encoding gradients for the reference (b0) acquisitions, which introduces a small but not negligible diffusion weighting to the “b0” images. This is taken into account during the tensor calculation.

Additional breath-hold CMR was performed to assess cardiac morphology and function in patients and controls. Retrospectively gated bSSFP cine sequences were acquired in 3 long-axis planes, followed by a contiguous stack of short-axis slices from the atrioventricular ring to the apex [26]. Late gadolinium enhancement (LGE) imaging for the assessment of regional fibrosis was also performed, using an inversion recovery prepared spoiled GRE.

### Image analysis

Ventricular volumes, function, mass, and ejection fraction was measured using a semi-automated threshold-based technique (CMRtools, Cardiovascular Imaging Solutions, London, UK), with indexing to body surface area [27]. The LV epicardial and endocardial borders were defined by manual planimetry excluding papillary muscles. Each short-axis slice was divided into 12 segments. The presence of LGE was recorded, and when present its extent was quantified using CMR42 software (Circle Cardiovascular Imaging, Calgary, Canada) with a full-width half-maximum threshold and expressed as percent of LV mass.

### Diffusion tensor analysis

All quantitative tensor information was post-processed with custom software, written in-house using MATLAB (Mathworks, Massachusetts, USA). In a first step, all the raw diffusion-weighted images were analyzed visually to reject frames corrupted by breathing motion or abnormal RR intervals. A minimum of two averages per direction, after frame rejection, was set as a requirement for inclusion in the study. The accepted frames were then co-registered to correct for intra-subject variations in breath-hold position (in-plane only) with a cross-correlation based algorithm for rigid displacement [28]. The subject’s RR-intervals during diffusion acquisition were then calculated from the acquisition times recorded in the DICOM files, and assumed to be half the time between frames (each frame runs over two heart-beats). This information was used to adjust the b-value for each image. A rank-2 diffusion tensor, and its eigensystem (set of three eigenvalues λ1, λ2, and λ3; and the respective eigenvectors: E1, E2, and E3) were then calculated for each voxel of the diffusion dataset. The eigenvalues and respective eigenvectors were sorted in a descending order according to the magnitude of the eigenvalues. A small percentage (3.2%) of the eigenvalues was negative due to motion, noise or mis-registration artifacts. This violates the assumption of a positive definite diffusion tensor, and therefore these values were averaged to the mean of neighbouring voxels.

**Figure 1:**
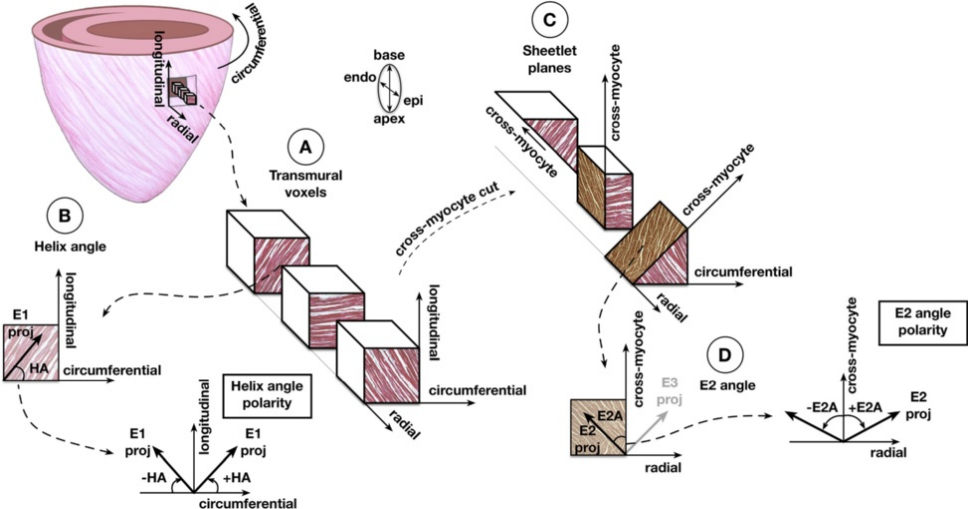
**Diagram of helix-angle and secondary eigenvector orientation.** Diagram illustrating how helix angle (E1A) and E2A values were calculated for each voxel. **A)** Cubic portions of three non-contiguous voxels are illustrated from subendocardial, mid-wall and subepicardial layers. The local cardiac coordinate directions, longitudinal, circumferential and radial, are marked. **B)** Helix-angle is calculated between the circumferential direction and the projection of the primary eigenvector, presumably parallel to myocytes, in the tangential plane shown. Examples of positive and negative helix angles are shown below. **C)** The cubes are each sectioned perpendicular to E1proj to calculate cross-myocyte components of diffusion, presumably constrained by the sheetlet and shear layer microstructure, which is shaded brown. **D)** The E2 angle is measured between the secondary eigenvector projection and cross-myocyte direction in the wall tangent plane. Examples of positive and negative E2 angles are shown on the right.

Two diffusion tensor measurements were calculated from the eigensystem: Helix-angle (HA), and secondary eigenvector angle (E2A) [8,29]. HA and E2A represent directional information from the first (E1) and second eigenvectors (E2) respectively. These were calculated by identifying the 3 orthogonal cardiac coordinate directions of each myocardial voxel: longitudinal, which is parallel to the left-ventricular long-axis and pointing towards the base; circumferential, which is tangential to the wall with a counter-clockwise direction when viewed from base to apex; and radial, which is given by the cross product of the previous two and pointing outward (Figure 1). The primary eigenvector E1 was then projected radially to the local wall tangent plane. HA was defined as the angle in this plane between the E1 projection and the circumferential direction in the range −90 to 90 degrees, being positive (right-handed helix) if rotated counter-clockwise from the circumferential as viewed from the outside, and negative (left-handed helix) if rotated clockwise. The cross-myocyte plane, perpendicular to E1, was then calculated for each voxel. Then E2 was projected onto this plane and E2A calculated in it, between E2 and the illustrated cross-myocyte direction. This angle was measured in the range [−90, 90], being positive if rotated clockwise from the cross-myocyte direction when viewed in the more circumferential direction, and negative if rotated counter-clockwise (Figure 1).

**Figure 2:**
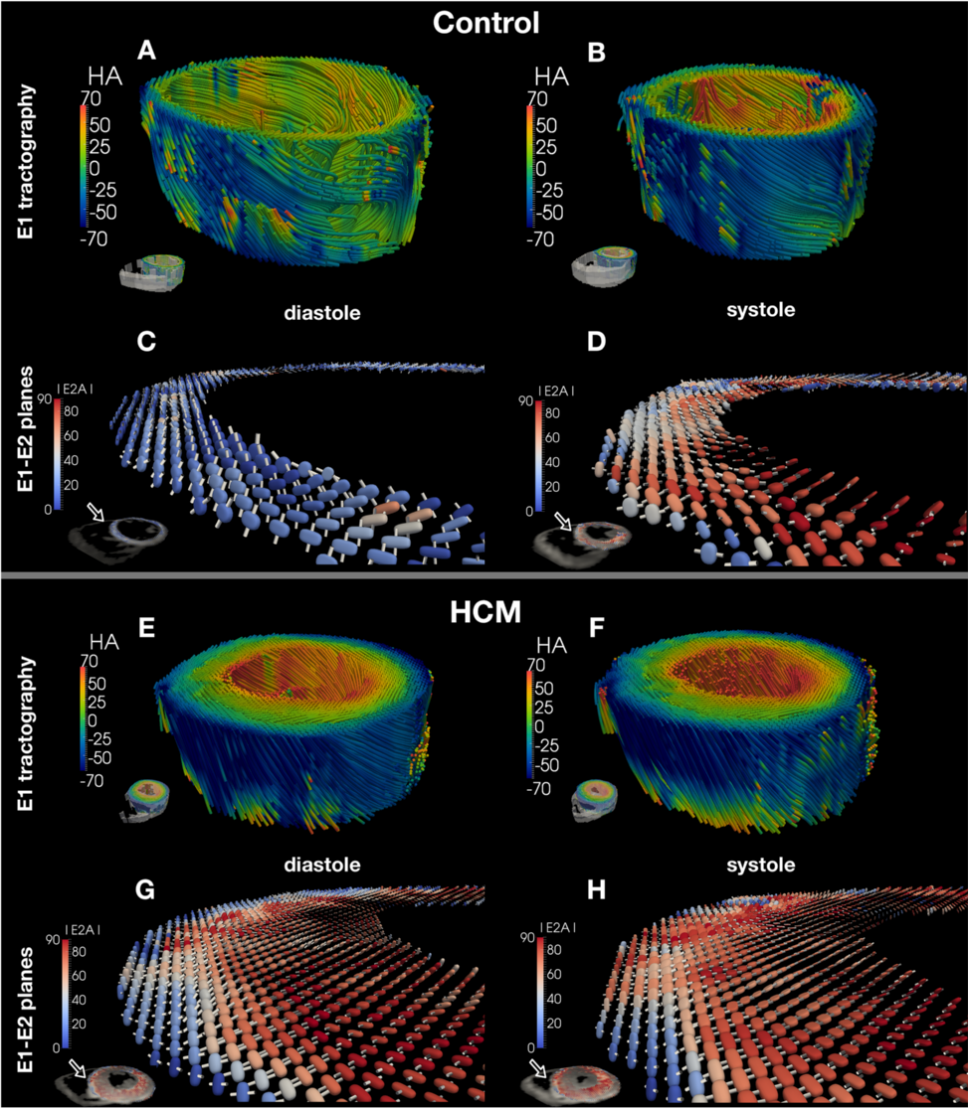
**3D visualization of helix-angle and secondary eigenvector orientation.** Three dimensional visualization of the principal myocyte-parallel (helix angle) and cross-myocyte (E2) directions of diffusion. Panels **A**, **B**, **E** and **F** show principal eigenvector tractography for a control and an HCM patient in late diastole and end systole respectively. Scale bars show color coding for helix angle. The poor quality of the tracts in the diastolic control example is due to the reduced spatial resolution available. Panels **C**, **D**, **G** and **H** show the diffusion tensor represented by superquadric glyphs superimposed with cylinders representing the E2 direction of each tensor only. The superquadric glyphs are color coded according to the absolute E2 angle as in the scale bars: blue towards wall-parallel and red towards wall-perpendicular. The glyphs typically reorientate from blue to red in the control, but in the hypertrophic septal regions in HCM, are aligned in what would normally be a relatively systolic, more wall-perpendicular orientation, in both diastole as well as systole.

For 3D visualization and comparison of E1 distributions, principal eigenvector tractography was performed, color coded according to helix-angle (Figure 2A,B,E,F). It must be emphasised that the obtained tracts are only used to aid on the visualisation of E1 orientation. No further analysis was performed on the tractograms, considering the limited number of slices acquired.

To display mean intra voxel sheetlet angulation in the septal and anterior wall regions, the full diffusion tensor was represented by superquadric glyphs, and superimposed with long thin white cylinders, which represent the orientation of E2. The tensors were color coded according to their absolute E2A value (Figure 2C,D,G,H). 3D visualization of tensor information and tractography was performed using Python and PARAVIEW (Kitware, NM, USA).

### Quantification and statistical analysis

The measured HA and E2A values were binned and plotted as histograms for all subjects in order to visualize the angle distribution. For further analysis of the effects of hypertrophy, E2A data was presented in a scatter plot. E2 mobility was defined as the change of mean absolute E2A values from diastole to systole. E2 mobility uses the absolute value of the angle so polarity is ignored to provide a measure of change of angulation relative to the local wall tangent plane from diastole to systole. Global E2A results included all LV segments in all 3 slices. For further insights in the HCM group, myocardial segments were categorized by consensus by 2 observers (12 segments per short-axis slice): segments with hypertrophy and LGE (H+LG+); segments with hypertrophy but no LGE (H+LG-); and segments with no hypertrophy or LGE (H-LG-). Segments were included in the H+ or LG+ categories even if hypertrophy or late-enhancement were only partially included. Mean absolute E2A values were compared between groups with the Mann–Whitney–Wilcoxon test. Statistical significance was considered present when p < 0.05 after Bonferroni correction for the number of tests performed.

## Results

### Patient characteristics

The baseline demographics and clinical characteristics of control and patient groups showed no significant differences (Table 2). The time spent in diffusion acquisition was 42 ± 7 min (mean ± SD) for controls, and 46 ± 7 min for HCM patients. The number of required breath-holds was 57 ± 4 for controls, and 63 ± 9 for HCM patients. The SD recorded on the RR interval, during diffusion acquisition, were 38 ± 9 ms for controls, and 41 ± 20 ms for HCM patients.

**Table 2:** **Baseline patient characteristics**

	**HCM Patients (n = 11)**	**Controls (n = 11)**	**P value**
Age (years)	62 ± 9	56 ± 10	0.16
Male	8 (73%)	6 (55%)	0.40
Body surface area (m^2^)	1.89 ± 0.2	1.83 ± 0.2	0.52
Body mass index (kg/m^2^)	26.1 ± 3.6	24.2 ± 2.3	0.15
RR interval (ms)	995 ± 150	984 ± 108	0.84
Mid systolic pause (ms)	373 ± 70	353 ± 41	0.42
Mid diastolic pause (ms)	798 ± 96	731 ± 79	0.09
Distance of mid image slice from apex (measured in systole as a proportion of total ventricular length)	0.53 ± 0.06	0.53 ± 0.04	0.74
Indexed LV EDV (mL/m^2^)	65 ± 15	78 ± 16	0.08
Indexed LV ESV (mL/m^2^)	14 ± 5	23 ± 5	0.001
LV ejection fraction (%)	79 ± 6	71 ± 3	<0.001
LV mass index (g/m^2^)	104 ± 41	67 ± 15	0.009
Maximum end-diastolic wall thickness (mm)	23 ± 4	9 ± 1	<0.001

(mean ± SD, or number (%) of patients).

Average LV mass, wall thickness and ejection fraction were greater in the HCM group and end-systolic volume was smaller. The ventricular morphology, risk factors for sudden death and LGE patterns for the HCM patients are summarized in Table 3. Ten out of 11 patients had asymmetrical septal hypertrophy and 1 patient had anterior wall hypertrophy. The median number of risk factors for sudden death was 0 (range 0–2). Six patients had LV outflow tract obstruction. All patients had regional fibrosis on LGE imaging usually affecting the septum and representing 9.1% of LV mass.

**Table 3:** **Morphology and late gadolinium enhancement in the 11 HCM patients**

**LV morphology & risk factors**	
Asymmetrical septal hypertrophy n (%)	10 (91%)
Anterior hypertrophy n (%)	1 (9%)
LV outflow tract obstruction at rest n (%)	6 (55%)
Number of risk factors for sudden death: median (range)	0 (0–2)
**Presence of late gadolinium enhancement**	11 (100%)
**Extent of late gadolinium enhancement (% of LV mass)**	9.1 ± 3.6%
**Location of gadolinium enhancement:**	
Septum	10 (91%)
Anterior	4 (36%)
Inferior	2 (18%)
Lateral	4 (36%)
> 1 region	8 (73%)

### Diffusion tensor visualization

Figure 2 shows 3-dimensional graphic representations of the orientations of E1 (mean intravoxel helix-angle) and absolute E2 (greater cross-myocyte direction of diffusion) rendered for 1 control and 1 HCM patient at both cardiac phases. The expected helix-angle distributions are observed, transitioning inwards transmurally from left-handed, through circumferential to right handed, without obvious changes of layer-wise orientation between phases, or between the control and the patient. In contrast, glyph representations of the diffusion tensor, and in particular orientation of E2, show unmistakable reorientation, reflecting phasic changes in absolute E2A. E2 tilts towards parallel to the wall-plane in diastole, and towards perpendicular in systole, particularly through mid-wall and subendocardial voxels, consistent with the predicted sheetlet reorientations during the cardiac cycle. In the HCM patient however, most obviously in the most hypertrophic wall region, the phasic changes are less pronounced, with the glyphs remaining relatively perpendicular to the local wall plane in diastole as well as in systole.

### Diffusion tensor quantification

**Figure 3:**
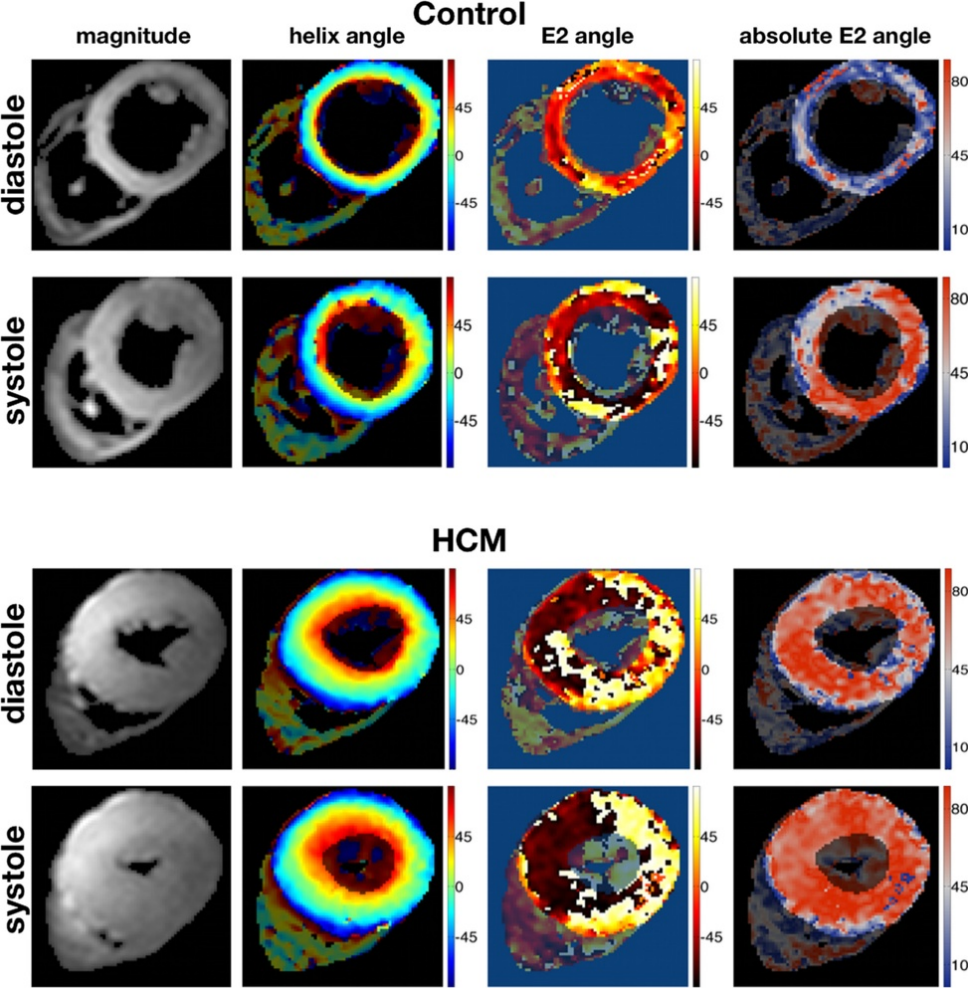
**Magnitude, helix-angle, and secondary eigenvector orientation maps.** Examples of an averaged magnitude image with the corresponding HA, E2A, and absolute E2A maps for a control and an HCM subject, at the two imaged cardiac phases. While positive and negative angles are colored differently in the E2A map to distinguish different dominant regional orientations in the individuals shown, maps of absolute E2A in the rightmost column allow clearer appreciation of angulations relative to the wall plane, changing from diastole to systole in the control, but remaining relatively steep or systole-like in both phases in HCM.

cDTI was successfully performed on all subjects. Only 3.2% of all eigenvalues were spuriously negative, for which we assigned the positive mean values of adjoining voxels. In total the number of myocardial voxels analyzed for each group was: controls-systole 28,836 voxels; controls-diastole 26,034 voxels; HCM-systole 45,791 voxels; HCM-diastole 44,661 voxels. The greater number of voxels in HCM patients occurred because of LV hypertrophy. The measured E2 angles were found to be meaningful (more details in Additional file 1: Appendix 1). An example of a mid-slice averaged magnitude image, together with the respective HA, E2A and absolute E2A maps at both cardiac phases is shown in Figure 3 for a control and an HCM patient.

**Figure 4:**
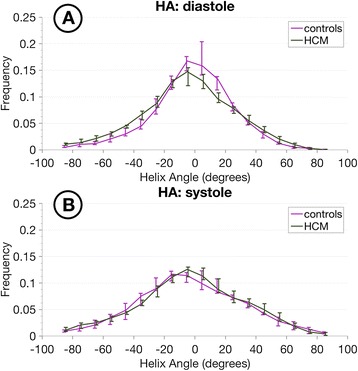
**Helix-angle histograms.** Histogram of myocardial HA values measured in all three slices per subject at the two cardiac phases for the two groups (bin size 10 degrees). The lines represent the median for each bin and the vertical bars the corresponding interquartile range. **A)** Diastole, **B)** Systole.

**Figure 5:**
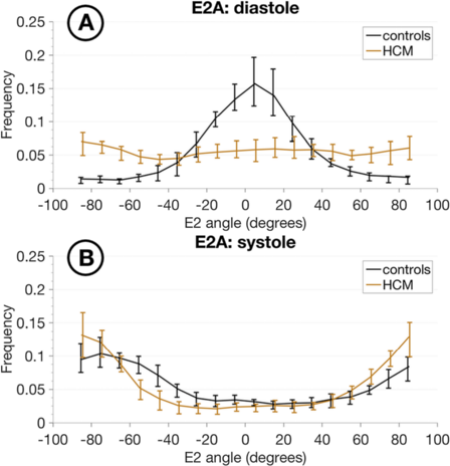
**Secondary eigenvector orientation histograms.** Histogram of myocardial E2A values measured in all three slices per subject at the two cardiac phases for the two groups (bin size 10 degrees). The lines represent the median for each bin and the vertical bars the corresponding interquartile range. **A)** Diastole, showing predominance of low angles, towards wall-parallel, in controls, and a relatively wide even distribution of angles from all wall regions in HCM. **B)** A predominance of high angles, towards wall-perpendicular for both groups, with more extreme angles towards wall-perpendicular for HCM.

The distribution of HA is shown in Figure 4. In general, HA maintained the same distribution between cardiac phases and also when comparing controls and HCM patients, all showing greatest frequency around the circumferential orientation (i.e. angles around 0 degrees). The main difference was in the controls, where diastolic distribution shows a narrower peak when compared to the systolic distribution. Contrasting with HA results, the E2A values did not follow an obvious spatial pattern within the myocardium, but showed substantial changes between the 2 cardiac phases, especially for controls (Figure 3). A histogram of the measured myocardial E2A values for both groups is shown in Figure 5. Systolic values peak at ±90 degrees for both groups, implying sheetlet orientations tilted in towards perpendicular to local wall planes, generally even more so in HCM than controls. However marked differences between the 2 groups were seen in diastole. In controls the diastolic angles peak at approximately 0 degrees, implying mean intravoxel sheetlet orientations towards parallel to local wall plane. However, in HCM, the diastolic distribution curves were flattened, showing a more homogeneous distribution across the range of angles without the tendency towards wall-parallel orientation seen in controls. The histograms were approximately symmetric, except for a slight predominance of negative systolic angles in controls.

**Figure 6:**
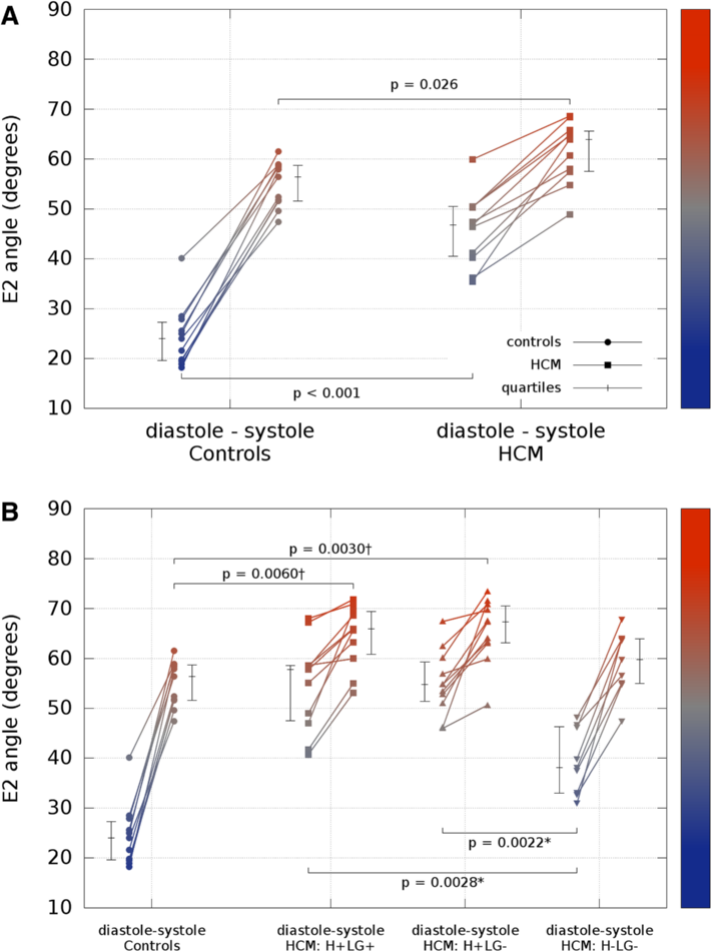
**Secondary eigenvector mobility.** Scatter plots showing the E2 mobility (mean absolute E2 angle change between diastole and systole) for all subjects at the two imaged cardiac phases. **A)** Global mean E2A values. **B)** Global controls vs HCM cohort with the myocardium divided into three different regions: regions with hypertrophy and LGE (H+LG+), regions with hypertrophy but no LGE (H+LG-), and regions with no hypertrophy or LGE (H-LG-). In all plots the median and interquartile range are shown. The plots include a colour bar, which encodes the y-axis values. Of note, it shows the most abnormal orientations, inclined steeply inward from the wall plane with low mobility, in the hypertrophic regions, whether not there is LGE evidence of fibrosis. *P-value multiplied by 2for Bonferroni correction for 2 tests. †P-value multiplied by 3 for Bonferroni correction for 3 tests.

Additional analysis of the E2A data is shown in the E2 mobility plots of Figure 6. Figure 6A shows the global mean absolute values for both cohorts at the 2 cardiac phases. There was a significant difference in global E2A in diastole between HCM and controls (p < 0.001). In systole, there was also a statistically significant difference between the 2 groups of lesser magnitude (p = 0.026). Figure 6B and Table 4 show further analysis of E2 mobility in the HCM cohort, with the myocardial absolute E2A values divided into 3 categories: segments with hypertrophy and LGE (H+LG+); segments with hypertrophy but no LGE (H+LG-); and regions with no hypertrophy or LGE (H-LG-). There was a significant difference in E2A at diastole between non-hypertrophic segments (H-LG-) and the other two groups H+LG+ and H+LG- (p = 0.0028 and p = 0.0022 respectively, both after Bonferroni correction for 2 tests). There was also a significant difference in E2A in systole between hypertrophic regions and controls for H+LG+ segments and H+LG- segments (p = 0.0060 and p = 0.0030 respectively both after Bonferroni correction for 3 tests).

**Table 4:** **E2A mobility**

	**E2A diastole median (** **°** **)**	**E2A systole median (** **°** **)**	**E2A Mobility (** ^**o**^ **)**
Global analysis			
Controls	24.0	56.4	32.4
HCM	46.8	63.9	17.1
p-values	<0.001	0.026	<0.001
Segmental analysis			
H+LG+	57.8	65.9	8.10
H+LG-	54.8	67.3	12.5
H-LG-	38.1	59.8	21.7
H+LG+ vs H-LG- p-values	0.0028*	0.16*	0.012*
H+LG- vs H-LG- p-values	0.0022*	0.096*	0.020*
Controls vs H+LG+		0.0060†	<0.001^†^
Controls vs H+LG-		0.0030†	<0.001^†^
Controls vs H-LG-		0.38†	0.0186^†^

*H* Hypertrophy, *LG* late gadolinium enhancement. *P-value multiplied by 2 for Bonferroni correction for 2 tests. ^†^P-value multiplied by 3 for Bonferroni correction for 3 tests.

**Figure 7:**
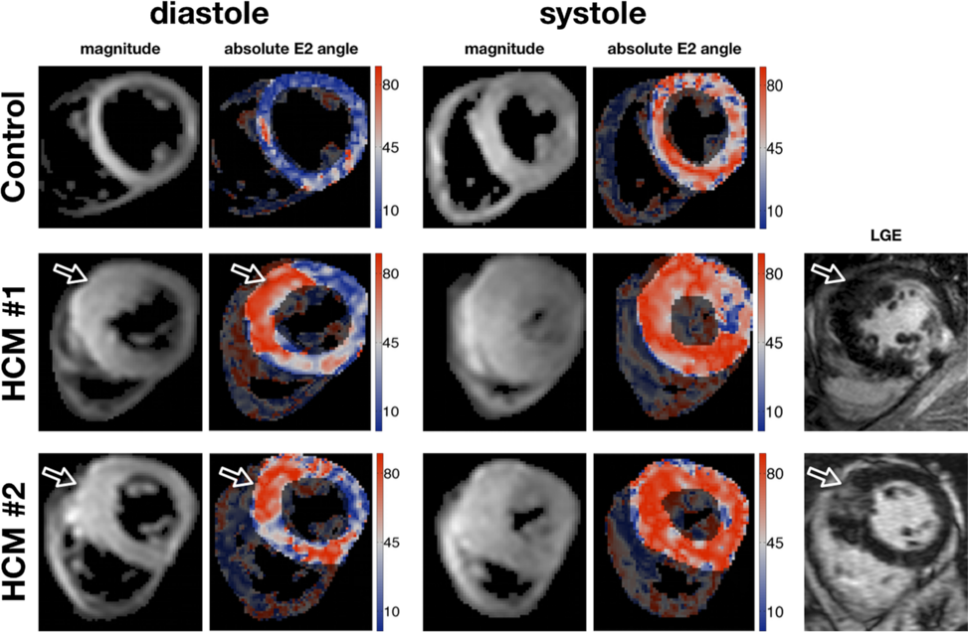
**Magnitude, secondary eigenvector orientation and LGE in HCM.** Averaged magnitude image and the respective absolute E2A angle maps for a control and 2 HCM examples with anteroseptal hypertrophy at the 2 imaged cardiac phases. Additionally the 2 HCM examples also have on the right the matching LGE images. E2A differences between controls and HCM can be seen in the hypertrophied regions, mainly in the diastolic phase. The non-hypertrophic lateral wall in both HCM hearts approaches the absolute E2A angles measured in the control heart.

Impairment of E2 mobility in hypertrophic segments is shown in Figure 7, where slices from 2 HCM hearts with asymmetric hypertrophy are compared with the corresponding slice from a control. The non-hypertrophic lateral wall in both HCM hearts approaches the absolute E2A angles measured in the control heart, while differences between the hypertrophic regions and the control values are clearly apparent in diastole. There was no obvious effect from the presence or absence of LGE on the E2A values in the hypertrophied anteroseptal wall, in accord with the results shown in Figure 6B.

## Discussion

**Figure 8:**
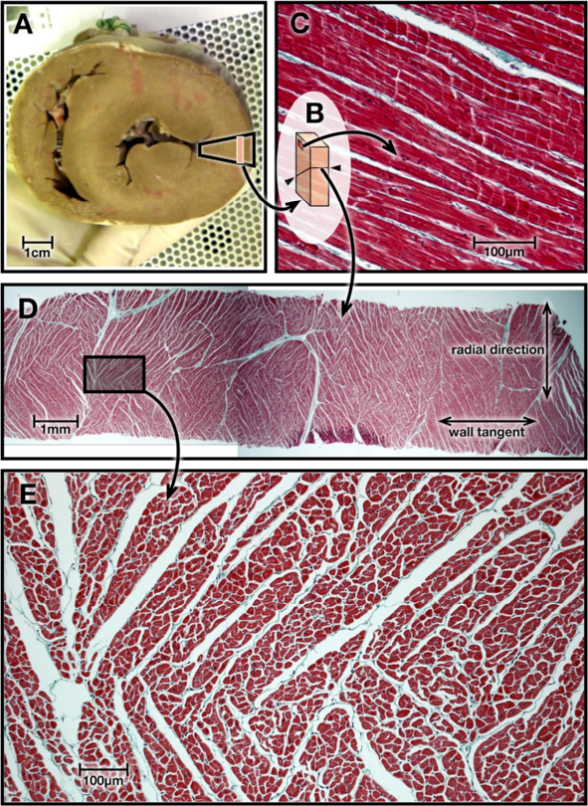
**Porcine heart histology.** The heart of a 60 kg pig that had been killed for food production, was excised, fixed by immersion in formalin and sectioned in a short axis plane **(panel A)**. A full thickness wedge, outlined in black, was cut from its lateral LV wall at mid ventricular level. From it, a slice about 2 mm thick was made by two cuts parallel to the local epicardial surface as indicated by the orange rectangle. This slice **(inset B)** was then cut obliquely, as indicated by lines between the arrowheads, perpendicular to the local myocytes. The two pieces were set in wax and selected surfaces sectioned by microtome and trichrome stained for histology. A high power image of part of a wall tangent face, indicated by the small red region in B, is shown in **panel C**. The long axes of myocytes lie nearly parallel to this slice and a number of pale Z bands between sarcomeres are visible. A low power image of a cross-myocyte plane is shown in **panel D**, giving an overview of laminar structures, which slope obliquely to its upper and lower wall tangent edges, generally in two different oblique populations. The upper edge is the more endocardial, corresponding to the line arrowed obliquely in B. The area indicated by the black rectangle is magnified in **panel E**, where the transected myocytes can be seen to be aggregated in sheetlets, 4–8 myocytes thick, separated by white fissures or shear layers. The scale bars allow the structures and textures seen in each panel to be considered in relation to the typical root mean square distance of aqueous diffusion of about 40-60 μm in a cardiac cycle, and to the dimensions of the cDTI voxels of 2.7 × 2.7 × 8 mm acquired in our study.

**Figure 9:**
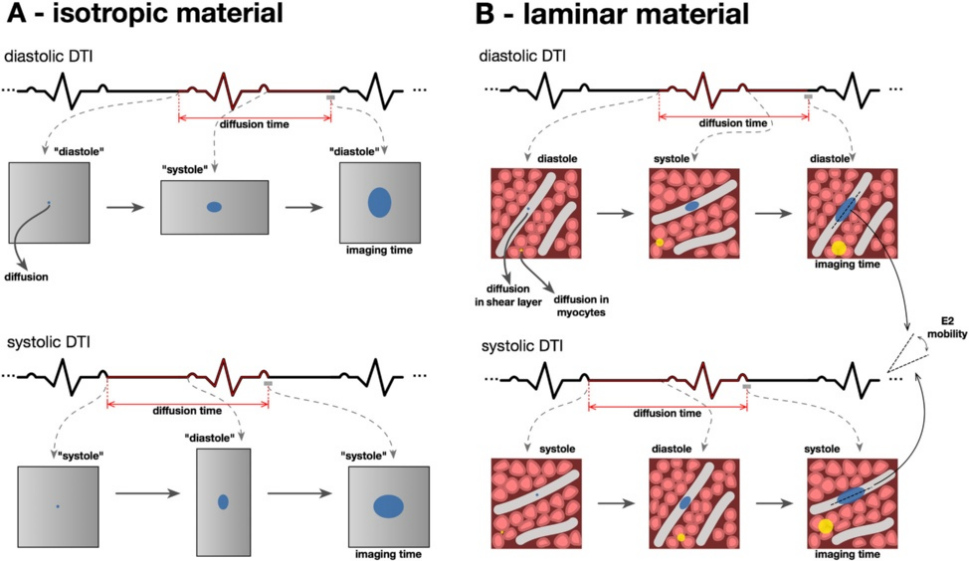
**Diffusion on isotropic and laminar materials. A**: Schematic illustration of the effects of a cycle of strain on aqueous diffusion through a structurally isotropic material such as a homogeneous gel. The expected progression of limits of diffusion from a single point is illustrated (blue ellipse). On the left panels the DTI sequence is initiated; the mid panels show the progress of diffusion at the opposite phase of the cycle; and the right hand panels show the extent of diffusion at the time of DTI readout. **B**: Schematic illustration of supposed aqueous diffusion through a laminar material. The changes between diastole and systole entail the shearing and slight swivelling of the sheetlets and shear layers. In general these maintain their proportions, but not their orientations. The anistropies of diffusion of water molecules through different parts of such a complex dynamic fabric remain unknown and hard to predict. However, it seems likely that cross-myocyte diffusion would extend most freely along shear layers (blue) and least freely through the myocytes (yellow) aggregated in a single sheetlet, as indicated very approximately by the two examples of diffusion boundaries. Importantly, cDTI is only, at best, capable of measuring averaged values of all diffusions in a voxel, including those along different populations of shear layers.

We believe this is the first report of *in vivo* systolic and diastolic measurements of the principal (E1) and second (E2) directional components of intramyocardial diffusion in HCM patients as measured by cDTI. The LV helix-angle (HA) distribution (Figures 3 and 4), which is individually and collectively consistent with previous *ex vivo* studies, showed only slight changes between systole and diastole [3,21], or between control and HCM hearts [24]. In contrast, we found marked changes between systolic and diastolic phases in the principal cross-myocyte direction of diffusion E2A, and these are consistent with previously reported changes of transmural angulation of the laminar sheetlet and shear layer structures [3,21,30]. We assume, as others have, that the measured cross-myocyte diffusions depend on the relative freedom of molecules to diffuse, particularly through the extracellular fluid of shear layers between sheetlets. The cyclic changes of E2 angle (E2A) measured in volunteers were in keeping with previously reported changes of transmural orientation of sheetlets and shear layers (Figure 6). However, interpretation of them is complicated by the need to consider previously postulated effects of myocardial strain on diffusion. As microstructures and micro-strains are likely to be fundamental to interpretation of cDTI, we include histological sections from a pig heart prepared in our own institution (Figure 8) and a schematic illustration (Figure 9). Together, these are intended to help conceptualise the possible effects of myocardial microstructures and their expected patterns of deformation on diffusion.

### The issue of strain

The microstructure of myocardium is not isotropic. However, with a view to its potential relevance to cDTI, Reese et al. considered the effects of cyclically applied strain on aqueous diffusion through a structurally isotropic material [31] They used an elastic gel phantom, periodically indented by a hemi-spherical driver. This caused radially directed compression of the gel with counter-stretching in the orthogonal circumferential directions, analogous with the radial thinning and bidirectional elongation of LV walls in diastole. On release, elastic restoration of the gel was associated with radially directed thickening and bidirectional shortening, analogous with systolic deformations of the LV wall, as imaged macroscopically. As predicted by the underlying theory of diffusion through an isotropic material during deformation, DTI in the ‘systolic’ phase showed anisotropy, with increase of the radial and reduction of the circumferential components of diffusion. We have schematically illustrated this effect in Figure 9A. The converse was found by DTI in the diastolic phase. Given normal, healthy myocardial strain, comparable strain-induced directions and amounts of anisotropy might be thought to account for the changes of E2A that we found in healthy volunteers. Strain might be taken as the underlying explanation, except for the following crucial considerations:

Firstly, the myocardium is not structurally isotropic, but orthotropic in most LV regions [9], meaning that it has different constituent properties in three orthogonal directions: the myocyte, sheetlet and sheetlet normal directions. Diffusion taking place through it is likely to be constrained by microstructural boundaries in ways that modify the postulated effects of strain on diffusion, particularly for the inherent long mixing-times (one heart-beat) of the STEAM sequence. Furthermore, the constituent microstructures undergo their own distinct small-scale deformations, including the shearing and cyclic reorientation of extracellular fluid in the interstitial shear layers. This is perhaps the most pertinent of several types of micro-deformation in contracting myocardium that may contribute to the measured anisotropies of diffusion. Others include the even smaller scale interdigitation of actin and myosin nanofilaments within sarcomeres, the lengthwise shortening and slight circumferential counter-thickening of whole myocytes and the reorientations of the sheetlets in which the myocytes are arrayed. These micro-deformations underlie but can, especially in the cross-myocyte directions, differ in magnitude and sign from deformations imaged macroscopically. The marked systolic thickening of myocardium is known to be caused more by laminar reorientations than by the thickening of individual myocytes [7]. This amount of radial thickening, combined with systolic shortening in cross-myocyte as well as myocyte directions in wall tangent planes, is only achievable through the presence and mobility of laminar microstructures.

Secondly, the effects of strain cannot account for the exaggerated systolic anisotropy of diffusion that we found in the hypertrophied regions of HCM hearts. As is well known in HCM, strain is reduced, especially in hypertrophic regions [20]. However, in systole, we measured above normal E2As implying marked predominance of radial over tangential components of diffusion. In diastole, on the other hand, in spite of the fact that even the hypertrophic myocardium had undergone radial thickening with slight longitudinal and circumferential shortening during systole, albeit of limited amount, there remained a predominance of radial over tangential components of cross-myocyte diffusion, with E2As greater than 45°. From the postulated effects of strain alone, the opposite would have been the case. To appreciate an alternative, more plausible interpretation of the HCM DTI results, the function of laminar microstructures should be considered further.

### Sheetlet and shear layer function

The contraction of myocytes of one myocardial layer, for example the sub-epicardial layer, imposes cross-myocyte shortening on the simultaneously contracting myocytes of a different layer, in this case the subendocardial. As mentioned in the introduction, it is this potential conflict that requires the presence and function of laminae: the sheetlets and shear layers not only offer compliance that allows cross-myocyte shortening in spite of their individual thickening, but the changing angulations of laminae, like the folds of a compressed concertina [7], contribute to wall thickening. The reorientations of laminae of a particular region are driven not by their own force, but rather by the contraction of deeper and/or more superficial myocytes of the same region. Contraction of myocytes through all depths of any given segment causes laminae to adopt steeper, through-wall obliquity, with increase of E2A values and of wall thickness. This underlies our interpretation of the elevated E2As found in the hypertrophic regions of HCM patients, in diastole as well as systole.

### HCM results

We had hypothesized that sheetlet mobility in HCM would be impaired, which we found. Our results also show that the diastolic orientations appear markedly abnormal, with the E2A of HCM hearts retaining relatively systolic conformation in diastole. This abnormality was associated with increased wall thickness, independent of the presence of myocardial LGE (Figure 6B). This suggests that the wall’s state of contraction or hypertrophy (not presence of LGE) is the dominant determinant of E2A in HCM, a finding that is consistent with findings by Aletras et al., that regional contractile heterogeneity in HCM could not be explained by the distribution of fibrosis [20]. It must however be noted that although capable of detecting regional fibrosis, LGE imaging is not suitable to image diffuse fibrosis [32]. T1-mapping is an emerging technique more suitable to detect and quantify diffuse fibrosis [33].

Although less marked than the diastolic abnormality, we also found exaggerated E2A values in systole, consistent with a hypercontracted state.

Our *in vivo* data, which suggests relatively systolic sheetlet orientations and impaired relaxation is consistent with current hypotheses of the pathophysiological consequences of HCM-causing mutations from biochemical studies and transgenic models. A common property of HCM-causing mutations is increased myofilament or myosin ATPase calcium sensitivity [34–37]. These mutations increase cardiomyocyte tension for a given cytoplasmic calcium level, particularly in the diastolic range, slowing relaxation kinetics, limiting diastolic relaxation, and under some circumstances increasing the tension generated during systole. Exaggeration and persistence of systolic tone, and delayed relaxation, have implications for myocardial perfusion given that coronary flow predominantly occurs in diastole enabled by myocardial relaxation [38], and can be compromised in HCM [39].

The improved understanding of pathophysiological mechanisms has potential implications for novel approaches to therapeutic intervention [40,41]. It may be feasible to use cDTI to identify abnormal myocardium, before the onset of fibrosis, and monitor its response to the effects a drug, known or novel, initially in animal experiments and, if apparently successful, ultimately in patients.

No macroscopic evidence of disarray in the HA maps of HCM patients was found. These findings contrast with the work of Tseng et al., which found more longitudinally oriented HAs in the hypertrophied epicardial septum when compared to the normal heart [42]. We consider there may be 2 factors, which have contributed to this difference. In some of the HCM subjects, longitudinal oriented HAs that were considered to be hypertrophy from the septo-marginal trabeculations of the right-ventricle were excluded from analysis of the left ventricle. Additionally our cohort of HCM subjects was relatively old with relatively few risk factors and might therefore have had limited myocardial disarray.

The spatial-resolution achievable *in vivo* cDTI is limited by acquisition parameters chosen for suitability in patients with limited breath-holding ability. In controls, diastolic wall thinning reduces effective resolution of differences between layers, although this was less problematic in HCM. A zero-filling interpolation algorithm was used. This has the potential to increase spatial resolution by using the corners of k-space, although the spatial resolution improvement becomes angle dependent [43]. The impact of this interpolation in measuring mean E2 angles was tested in 18 datasets acquired for another study; results showed minimal differences of less than 3 degrees when compared to no interpolation. A maximum difference lower than 8 degrees was found for the helical-angle. We therefore expect this interpolation to have minimal impact in the diffusion tensor calculation.

### Limitations

There are a number of limitations to this study. As discussed above, the sensitivity to myocardial strain of the cDTI technique remains unclear. It is likely to differ from that of a homogenous, elastic gel and further work is required to investigate whether or not it contributes to the results reported.

The relation between E2A and the orientations of sheetlets within a voxel is unlikely to be straightforward. Thousands of cardiomyocytes arrayed in hundreds of sheetlets, with potentially two or more opposing populations may occupy a single 2.7 × 2.7 × 8 mm^3^ imaging voxel [40,44]. The sheetlets are expected to change orientation through the cycle relative to one another as well as to the local wall plane. Angles between adjacent sheetlet populations of the mid layer might, for example, if viewed like the folds of an accordion, be obtuse when extended longitudinally in diastole, but acute when brought together in systole. Were a voxel to contain 2 equal opposing populations, this might result in very small, almost wall parallel, E2As in diastole even though individual sheetlet populations remained oblique. Interestingly, however, our analysis of E2A supports the predominance of one orientation of sheetlet population in most voxels. We have not yet identified any consistent pattern of distribution of the ‘positive’ and ‘negative’ slopes of these between individuals. The different and unknown orientations of 2 or more sheetlet populations in a voxel cannot be elucidated using a single tensor. cDTI techniques with higher angular diffusion resolution (multi-tensor, diffusion spectrum imaging, and q-ball) would be needed for this [45], although the acquisition times required would currently be prohibitive in a clinical setting.

Myocardial coverage was limited to 3 slices. The lack of whole heart coverage made it challenging to define the longitudinal wall tangent direction. In this work, it was taken as perpendicular to the short-axis plane, which is a better approximation for the basal and mid than the more apical slice. Intra-subject variations in breath-hold are corrected with a rigid in-plane registration algorithm; through-plane variation remains uncorrected. The SPAMM imaging was used to track manually the imaged slices from systole to diastole, with the objective of reducing differences in the imaged volume between the two phases. This method is very approximate and it does not take into account the complex cardiac deformation present entirely, particularly in the HCM cohort. Nevertheless we believe this method to be sufficient for the DTI metrics shown, as we are only showing differences in global mean values.

The diffusion-encoding scheme used had only 6 directions. Recent DTI work in the brain showed comparable Mean Diffusivity (MD) and Fractional Anisotropy (FA) between 6 directions and 30 or 60 direction schemes (acquisition time kept constant) [46]. DTI numerical simulations also show comparable tensor accuracy when trading directions for averages [47]. It remains unknown if 6 direction schemes are comparable to higher order schemes for cDTI. A recent study from our group shows that both HA and E2A maps become smoother with higher b-values (>750 s/mm^2^). However E2A global mean values do not change substantially [48].

The averaging of non-positive definite tensors was performed for the eigenvalues directly. We appreciate that this is not a robust method, it maintains the neighbouring FA and MD, but it lacks sophisticated tensor interpolation. Nevertheless it affects only a small percentage (3.2%) of the eigenvalues measured, so we expect the impact to be small for the global means of E2A.

## Conclusions

*In vivo* cDTI in normals delivered apparently meaningful measurements of cross-myocyte diffusion, in keeping with previously reported myocardial sheetlet and shear layer reorientations from more wall-parallel in diastole to more wall-perpendicular in systole. In HCM, impaired diastolic reorientation of sheetlet populations was identified which was related to increased wall thickness and not LGE. The apparent persistence in diastole of a relatively systolic conformation in HCM may represent a novel *in vivo* human pathophysiological finding and would be consistent with biochemical studies and transgenic models that point to increased calcium sensitivity and impaired diastolic relaxation associated with known causative mutations.

Although the extent of the strain contribution to these results is currently uncertain, we believe that the measurements described in this study may represent a step towards *in vivo* investigation of myocardial structural dynamics.

The potential of this novel non-invasive approach to assess HCM pathophysiology *in-vivo* at the tissue level, combined with advances in the understanding of underlying molecular mechanisms, has potential for the development and evaluation of novel therapeutic approaches for patients with HCM.

## Additional file

Additional file 1:
**Appendix 1.**
