# Supplementary material for: In vivo cardiovascular magnetic resonance diffusion tensor imaging shows evidence of abnormal myocardial laminar orientations and mobility in hypertrophic cardiomyopathy
Source: J Cardiovasc Magn Reson. 2014 Nov 12;16(1):87. doi: 10.1186/s12968-014-0087-8 (PMC4229618; doi:10.1186/s12968-014-0087-8)
Supplement: Additional file 1: — Appendix 1. [file 12968_2014_87_MOESM1_ESM.doc]

**Appendix 1**

**Supplemental Methods**

In each voxel, E2 could be assigned its direction only if it differed in magnitude from E3. To check how frequently and clearly this was the case, histograms of the ratio of the third and the secondary eigenvalue, known as transverse anisotropy (TA), were calculated for all myocardial voxels. E2 can only be separated from E3 if TA is smaller than one. The angle E2A is calculated in the plane perpendicular to the first eigenvector, therefore the distribution of the calculated angles can be compared directly to a uniformly pseudorandom numbers distribution in a similar fashion to the work by Helm et al. [1] Bonferroni-corrected two sample Kolmogorov-Smirnov tests were used to compare both distributions in each subject in order to warrant E2 data significance.

The main eigenvector E1 orientation was analyzed in the main text by measuring its helix angle. Additional information can be retrieved by the transverse angle (E1-TA), which measures the deviation of E1 from the wall plane, i.e. it measures how parallel E1 is to the local wall plane. This was done by projecting E1 into the local radial-circumferential plane, and measuring the angle with the wall.

The E2A distribution as a function of transmural position in diastole and systole, in normals and HCM patients was also performed. The myocardium was divided transmurally in three regions with equal transmural thickness: Endo, meso, and epicardial.

DTI scalar maps of Helical angle (HA), E2 Angle (E2A), E1 Transverse Angle (E1-TA), and Fractional Anisotropy (FA) maps; and the corresponding LGE images, for three HCM subjects, not shown in previous Figures, were assembled as additional examples.

**Supplemental Results**

The histograms of the transverse anisotropy are shown in Supplemental Figure 1. In general the secondary eigenvalue is clearly distinguishable from the tertiary eigenvalue, with transverse isotropy above 0.9 for only 4% (SD 2%) of all voxels. The Kolmogorov-Smirnov comparison between the distribution of E2A values and a uniformly distributed pseudorandom numbers distribution rejects the null hypothesis that the measured E2A values are uniformly random for all datasets at a 5% significance level (Bonferroni-corrected).

The histograms of the transverse angle are shown in Supplemental Figure 2. In agreement with previous ex vivo measurements, E1 is generally parallel to the wall, with only a small percentage of voxels with angles bigger than 35 degrees. [2]

The E2A transmural distributions are shown in Supplemental Figure 3. In general the mesocardium region contains to a certain extent higher E2A values when compared to the endocardium or epicardium. Although the limited spatial-resolution available, with possible partial volume artefacts at the edges, must be taken into consideration when interpreting these results.

Supplemental Figure 4 shows HA, E2A, E1-TA , and FA maps for both diastole and systole, along with the corresponding LGE image for three HCM subjects not shown in previous Figures.

**Supplemental Figures and Figure Legends**


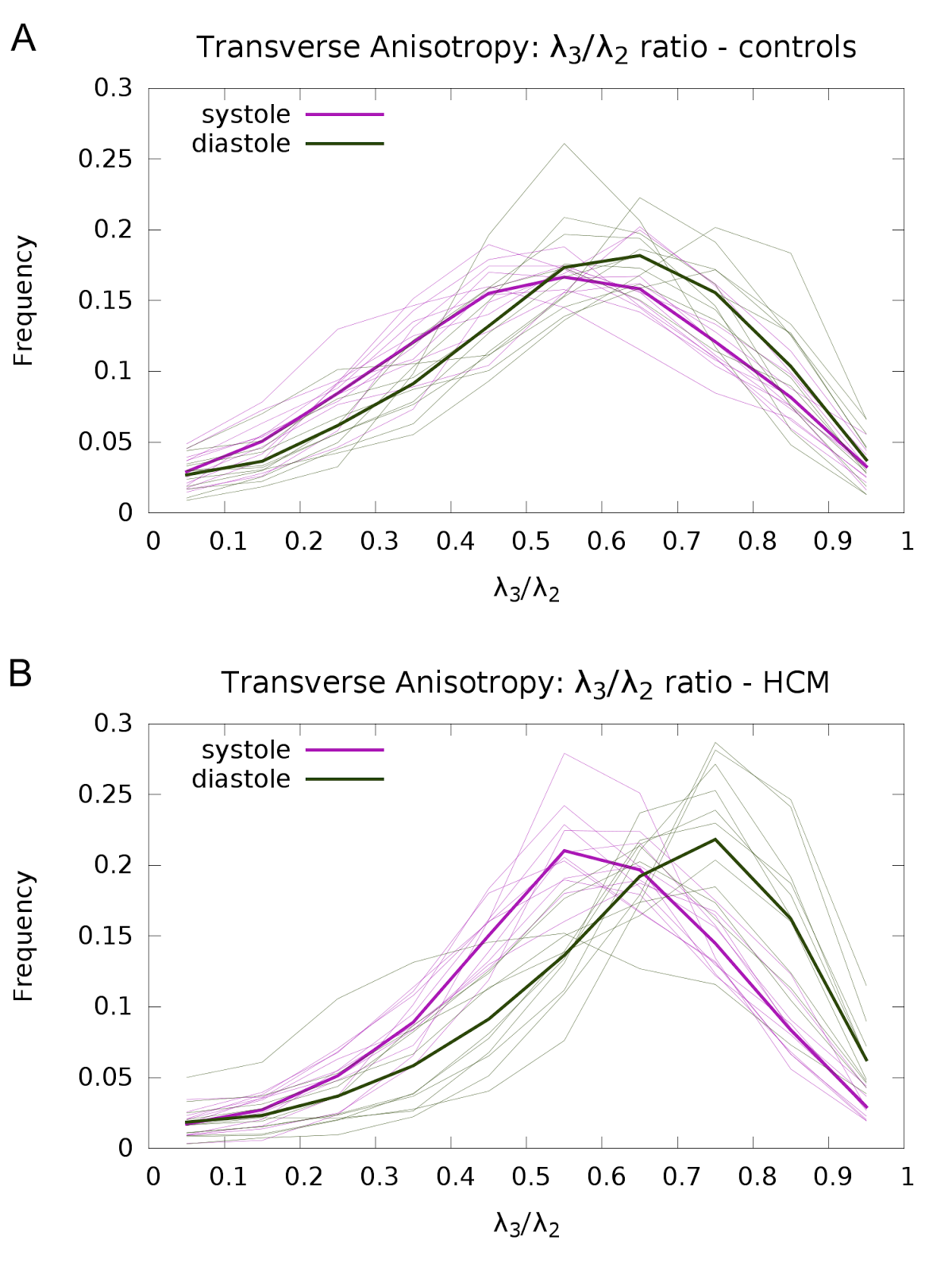


Supplemental Figure 1: Histogram of myocardial Transversal Anisotropy values measured in all three slices per subject at the two cardiac stages for the two groups (bin size 0.1). a) controls, b) HCM. The thin lines represent each individual subject, the thick lines the inter-subject average.


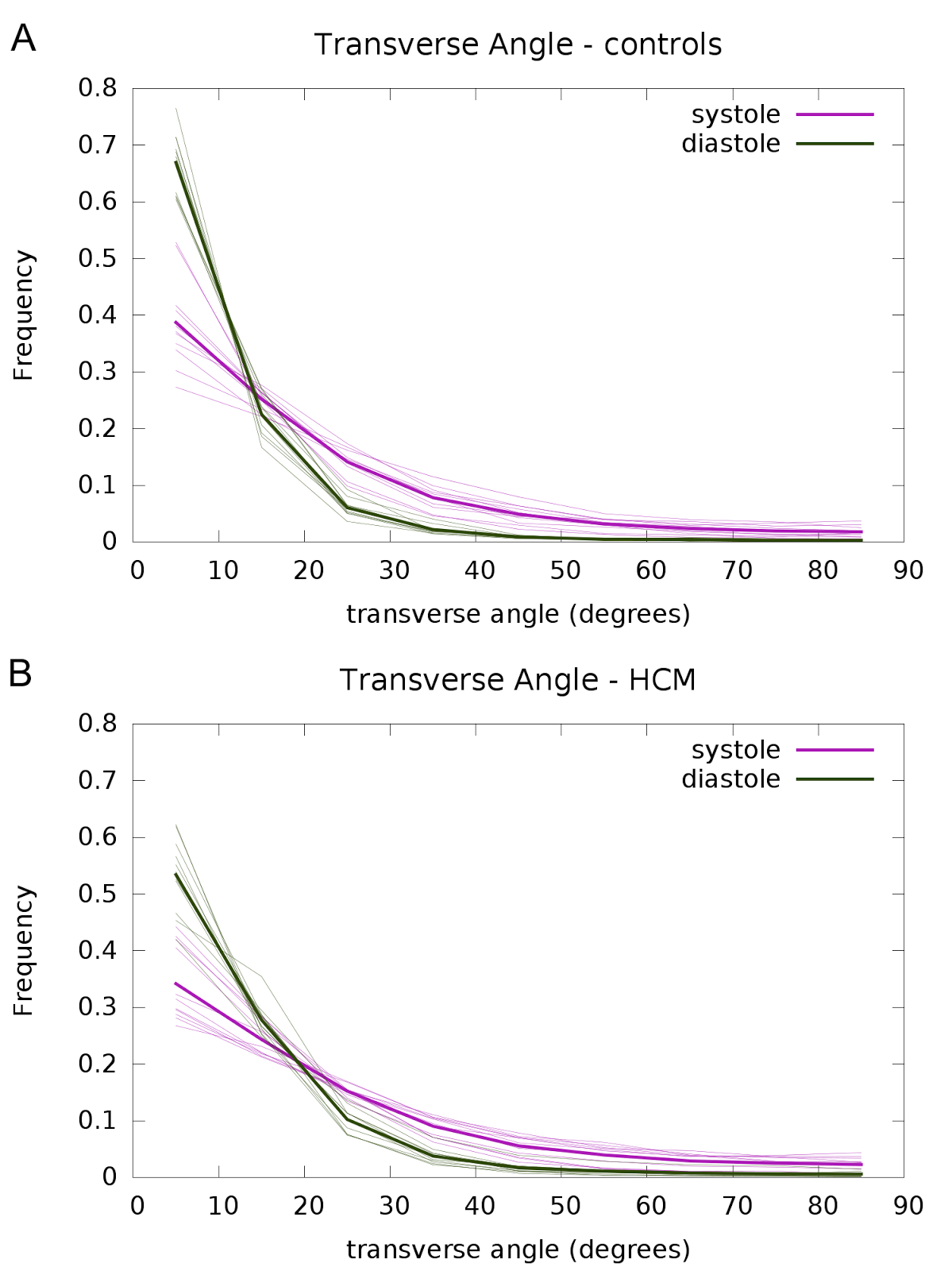


Supplemental Figure 2: Histogram of myocardial Transverse Angles in all three slices per subject at the two cardiac stages for the two groups (bin size 10). A) controls, B) HCM. The thin lines represent each individual subject, the thick lines the inter-subject average.


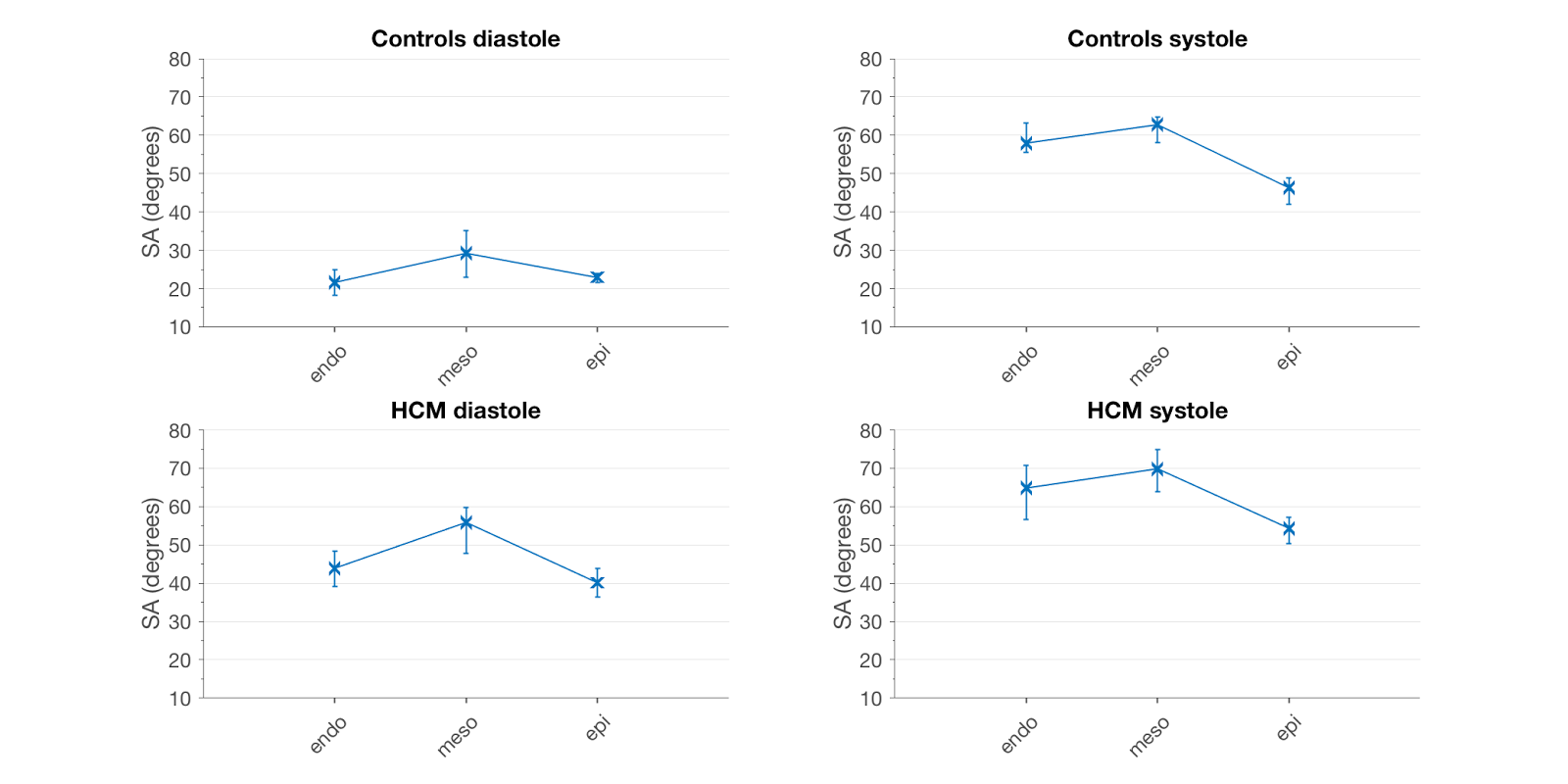


Supplemental Figure 3: E2A transmural distribution for both controls and HCM cohorts at systole and diastole. Each point represents the median and the interquartile range across subjects for each region.


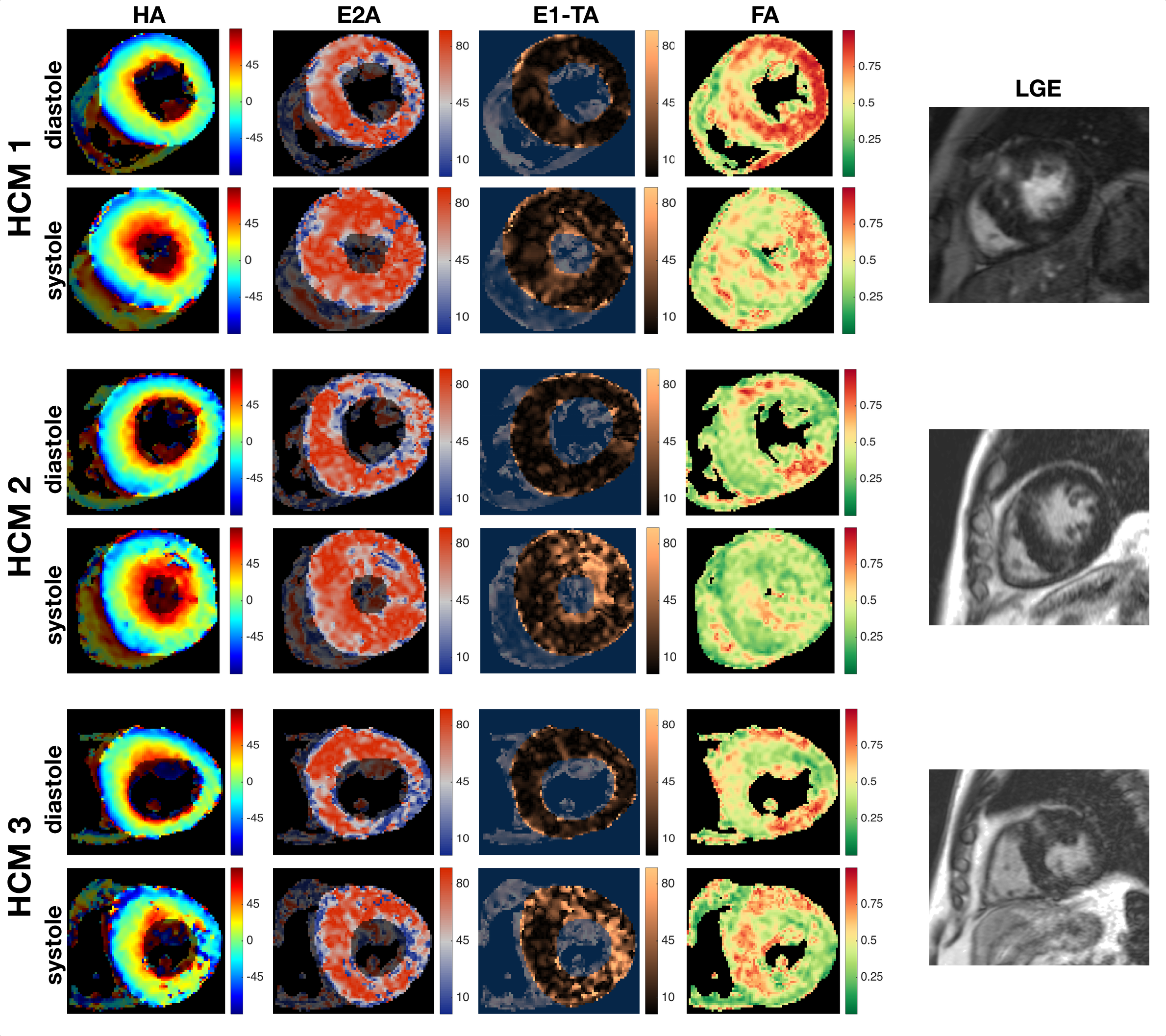


Supplemental Figure 4: Three HCM subjects showing: DTI maps of Helical Angle (HA), E2 angle (E2A), E1 Transverse Angle (E1-TA), and Fractional Anisotropy (FA) for both diastole and systole. The corresponding slice LGE image is also shown.

**Supplemental references**

[1] Helm PA, Tseng HJ, Younes L, McVeigh ER, Winslow RL. Ex vivo 3D diffusion tensor imaging and quantification of cardiac laminar structure. Magn Reson Med 2005; 54: 850–9.

[2] Lombaert H, Peyrat JM, Croisille P, Rapacchi S, Fanton L, Cheriet F, Clarysse P, Magnin I, Delingette H, Ayache N. Human atlas of the cardiac fiber architecture: study on a healthy population. IEEE Trans Med Imaging 2012; 31: 1436–47.
